# Brain Functional Alteration at Different Stages of Neuropathic Pain With Allodynia and Emotional Disorders

**DOI:** 10.3389/fneur.2022.843815

**Published:** 2022-05-02

**Authors:** Ya-Nan Zhang, Xiang-Xin Xing, Liu Chen, Xin Dong, Hao-Tian Pan, Xu-Yun Hua, Ke Wang

**Affiliations:** ^1^Acupuncture Anesthesia Clinical Research Institute, Yueyang Hospital of Integrated Traditional Chinese and Western Medicine, Shanghai University of Traditional Chinese Medicine, Shanghai, China; ^2^Department of Rehabilitation Medicine, Yueyang Hospital of Integrated Traditional Chinese and Western Medicine, Shanghai University of Traditional Chinese Medicine, Shanghai, China; ^3^Engineering Research Center of Traditional Chinese Medicine Intelligent Rehabilitation, Ministry of Education, Shanghai, China; ^4^Department of Traumatology and Orthopedics, Yueyang Hospital of Integrated Traditional Chinese and Western Medicine, Shanghai University of Traditional Chinese Medicine, Shanghai, China

**Keywords:** neuropathic pain, allodynia, negative emotions, functional magnetic resonance (fMRI), ALFF, DC

## Abstract

Neuropathic pain (NeuP), a challenging medical condition, has been suggested by neuroimaging studies to be associated with abnormalities of neural activities in some brain regions. However, aberrancies in brain functional alterations underlying the sensory-discriminative abnormalities and negative emotions in the setting of NeuP remain unexplored. Here, we aimed to investigate the functional alterations in neural activity relevant to pain as well as pain-related depressive-like and anxiety-like behaviors in NeuP by combining amplitude of low frequency fluctuation (ALFF) and degree centrality (DC) analyses methods based on resting-state functional magnetic resonance imaging (rs-fMRI). A rat model of NeuP was established *via* chronic constriction injury (CCI) of the sciatic nerve. Results revealed that the robust mechanical allodynia occurred early and persisted throughout the entire observational period. Depressive and anxiety-like behaviors did not appear until 4 weeks after injury. When the maximum allodynia was apparent early, CCI rats exhibited decreased ALFF and DC values in the left somatosensory and nucleus accumbens shell (ACbSh), respectively, as compared with sham rats. Both values were significantly positively correlated with mechanical withdrawal thresholds (MWT). At 4 weeks post-CCI, negative emotional states were apparent and CCI rats were noted to exhibit increased ALFF values in the left somatosensory and medial prefrontal cortex (mPFC) as well as increased DC values in the right motor cortex, as compared with sham rats. At 4 weeks post-CCI, ALFF values in the left somatosensory cortex and DC values in the right motor cortex were noted to negatively correlate with MWT and exhibition of anxiety-like behavior on an open-field test (OFT); values were found to positively correlate with the exhibition of depressive-like behavior on forced swimming test (FST). The mPFC ALFF values were found to negatively correlate with the exhibition of anxiety-like behavior on OFT and positively correlate with the exhibition of depressive-like behavior on FST. Our findings detail characteristic alterations of neural activity patterns induced by chronic NeuP and underscore the important role of the left somatosensory cortex, as well as its related networks, in the mediation of subsequent emotional dysregulation due to NeuP.

## Graphical Abstract

Brain functional alteration at different stages of neuropathic pain. 
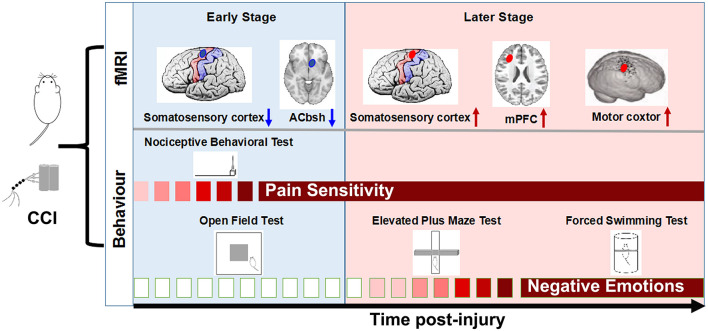

## Introduction

Neuropathic pain (NeuP), caused by pathology of the somatosensory nervous system, is commonly seen as a chronic condition in clinical practice (1). The general prevalence of NeuP is estimated to reach 7-8% and, as such, significantly decreases the quality of life and imposes a high societal burden (2–4). In addition to hyperalgesia, allodynia, and spontaneous pain, NeuP is also accompanied by the manifestation of emotional disorders including anxiety and depression (1, 5). Notably, emotional disbalance further exacerbates NeuP (6, 7). Although NeuP has become a major worldwide public health concern (8), the mechanisms underlying its pathogenesis remain unclear, and thus its treatment remains challenging.

The brain is involved in the central regulation of pain and the generation of negative emotions (9, 10). Noxious stimulation affects multiple brain regions, including the primary somatosensory cortex (S1), the secondary somatosensory cortex (S2), anterior cingulate cortex (ACC), prefrontal cortex (PFC), thalamus, nucleus accumbens (NAc), amygdala, and periaqueductal gray (PAG) (10, 11). Most of these aforementioned brain regions similarly engage in the regulation of emotion (12–16). As such, pain induces “an unpleasant sensory and emotional experience” and provides a physiological, structural, and functional basis for multi-dimensional changes seen in the setting of chronic pain (17). Previous studies have reported that the structural and functional disorders of these relevant brain regions contributed to the generation and maintenance of allodynia and negative emotions in chronic pain (18, 19). Multiple lines of evidence have suggested that obvious pathological pain develops early and that negative emotions do not become apparent until later (20). Furthermore, a recent study reported that pathological pain resulting from different etiologies affects distinct neural circuits (21). Whether a similar sequence of alterations in distinct brain regions corresponds to the manifestation of pathological pain and negative emotions in different stages of NeuP, however, remains unknown.

Functional magnetic resonance imaging (fMRI), a non-invasive neuroimaging technique, is used to evaluate the relationship between hemodynamic response and neuronal activity (22). It reflects both physiological functions and metabolic alterations caused by local neuronal activity *via* changes in blood-oxygen-level-dependent signal (BOLD) (23). As rs-fMRI can macroscopically detect resting-state neuronal activity, it is frequently used in the study of a variety of neuropathologies (24, 25). Brain diseases are associated with abnormal local spontaneous neuronal activity (26). Many methods have been exploited to characterize local properties of the rs-fMRI signal, including the amplitude of low-frequency fluctuation (ALFF) and degree centrality (DC) (26, 27). ALFF is used as an indicator to characterize the intensity of neural activity at a voxel, while DC reflects the intrinsic functional connectivity between a node (voxel) and other nodes within the brain, and can be used to evaluate hub nodes (27, 28). Here, rs-fMRI was used to investigate brain abnormalities in both the early and late stages of NeuP using ALFF and DC to assess relevant central mechanisms.

## Materials and Methods

### Animals

**Figure 1:**
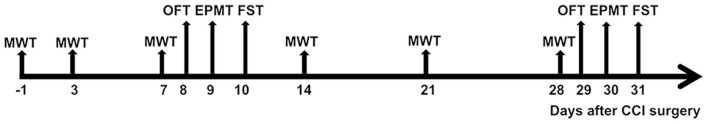
Schematic of the experimental timeline.

Male Sprague-Dawley (SD) rats (160-180 g) were purchased from Shanghai Slack Laboratory Animal Ltd. (Shanghai, China). The animals were kept under a 12/12 h reverse light cycle in a controlled environment at a temperature of 21 ± 1°C and relative humidity of 60–70%. Food and water were available to the rats at all times, and the animals were provided a 7-day acclimatization period to their new environment prior to experimentation. A total of 16 rats were randomly divided into sham (*n* = 8) and chronic constriction injury (CCI; *n* = 8) groups. Experimental procedures stated in the National Institutes of Health Guidelines for the Use of Laboratory Animals were approved by the Institutional Animal Care Committee of YueYang University of Traditional Chinese Medicine, Shanghai, China. All behavioral tests were performed between 8:00 am and 11:30 am. The flowchart of the experimental design is shown in Figure 1.

### CCI Model

Rats were anesthetized *via* intraperitoneal injection with sodium pentobarbital (50 mg/kg). Left-sided CCI of the sciatic nerve was achieved as previously described by us (20). A surgical incision of one centimeter was initially made in the middle of the thigh and the left sciatic nerve was exposed after being bluntly separated from the muscle. The exposed sciatic nerve was subsequently ligated in four passes using a gut suture (3-0 silk). In sham group rats, only the left sciatic nerve was exposed without ligature. All surgical procedures were performed by the same individual to prevent potential bias. All efforts were made to minimize animal suffering, and there were no rat deaths during surgery to establish CCI.

### Behavioral Tests

#### Nociceptive Behavioral Test

The von Frey plantar aesthesiometer (IITC, Woodland Hills, CA, USA) was used to measure mechanical withdrawal thresholds (MWT). Animals were placed separately in Plexiglas cages on a punching table for 15 min to allow acclimatization to the environment prior to testing. Each rat's left hind paw was stimulated three times at 5 min intervals during the formal examination. Paw withdrawal, flinching, or licking was regarded as positive behavior (29). Each value was recorded; MWT were represented by the mean values.

#### Open Field Test

The open-field test (OFT) was conducted to measure athletic ability and anxiety-like behavior (30). Rats were provided an acclimatization period of 30 min in the behavior assessment room prior to experimentation. The dimensions of the testing apparatus were 100 cm (length) × 100 cm (width) × 40 cm (height); it contained non-reflective black walls and floor. Each rat was gently placed in the central zone and allowed to freely explore the area for 10 min in a quiet environment. The central zone was defined as an area covering 40% of the total area of the box. SMART 3.0 software (Panlab, Cornella, Spain) was used to record and analyze time and distance traveled in the central zone, as well as the total distance traveled. The apparatus was cleaned after testing each rat using 75% ethanol.

#### Elevated Plus Maze Test

The elevated plus-maze test (EPMT) was used to measure anxiety associated with open spaces and height (31). The maze comprised two 50 × 10 cm open arms, two 50 × 10 cm closed arms, and a 10 × 10 cm central area. The closed arms were contained by boards 40 cm high. The maze was placed 80 cm above the floor in a testing room. Each rat was placed onto the central area facing one open arm and allowed to explore the maze for 10 min (20). Time spent in the arms of the maze and total distance traveled by rats was analyzed using SMART software. After testing each rat, the apparatus was cleaned as described above.

#### Forced Swimming Test

The forced swimming test (FST) was conducted to assess depressive behavior. Rats were placed into a glass cylinder (height 30 cm; diameter 18 cm) filled with water (23 ± 1°C) for 6 min. Immobility time throughout the 4 min of the testing session was evaluated. Immobility was defined as behavior manifesting by the rat only keeping its head above water and attempting to float with minimal exertion (32). The experimenter was blinded to both the CCI and sham groups.

### fMRI Acquisition

We performed fMRI scans at 11 and 32 days post-CCI and collected imaging data using a Bruker 7T magnetic resonance system (Bruker Corporation) with a coil. Before data acquisition, rats were anesthetized with 2.5% isoflurane and Medetomidine (0.025 mg/kg). Under continuous 1.5–2% isoflurane anesthesia, rats were fixed on the scanner with their heads immobilized. Body temperature and respiration were continuously monitored. Imaging data were obtained with the following interlayer scanning echo-plane parameters: interleaved scanning order; flip angle = 90°; slice thickness = 0.3 mm;, repetition time = 3,000 ms; 200 times points; imaging duration = 10 min; echo time = 20 ms; number of averages = 1; field of vision = 32 × 32 mm^2^; matrix = 64 × 64 voxels.

### fMRI Data Preprocessing

Data preprocessing was conducted using the Statistical Parametric Mapping 12 toolbox (http://www.fil.ion.ucl.ac.uk/spm/) based on the MATLAB 2014a platform. To match data to human dimensions, the first five time points were removed and images expanded by 10 × 10 × 10. This amplification procedure only modulated the dimension descriptor fields in the file header instead of changing interpolation. Manual stripping of none-brain tissue was then performed before further preprocessing. To minimize the temporal bias of slice acquisition, slice scan time correction was performed. To reverse the dislocation of voxels caused by head motion, spatial adjustment with rigid-body transformations was applied. The standard brain template in Schwarz's study was used to accomplish the normalization of common space, the voxel size for normalized images was 2.06 × 2.06 × 2 mm (33). Images were subsequently smoothed by a full width at half maximum quadruple as the voxel size (6.18 × 6.18 × 6 mm). For further preprocessing, temporal bandpass filtering (0.01–0.08 Hz) was applied to decrease the low-frequency drift based on the removal of covariates and linear trends.

### ALFF Calculation

ALFF depends on the blood oxygen level (BOLD) signal of each voxel and reflects the extent of spontaneous neuronal activity (20). Here, we calculated ALFF values for the traditional low-frequency band (0.01–0.08 Hz) and divided them by the global mean ALFF value within the brain mask (28).

### DC Calculation

DC represents the extent of interconnectivity between a given voxel (node) and other voxels, thus detailing the importance of the voxel or brain area. A change in the DC value of a node indicates altered connectivity; these changes were calculated during analyses (34). The DC was calculated using the REST toolbox (http://www.restfmri.net). A whole-brain functional connectivity matrix based on a REST-supported binary mask was constructed using Pearson's correlation coefficients between gray matter voxel. To improve normality and derive the Z-score matrix, the Fisher transformation was used. Regional functional connectivity strength was calculated as the sum of all the connections (*Z*-values) between voxels. Greater strength values were extracted and analyzed.

### Statistical Analyses

Statistical analyses were performed using SPSS 19.0 (IBM Corp., Armonk, USA). Behavioral data were expressed as the mean ± standard error (SEM). Differences in MWT between the two groups at multiple time points were compared *via* two-way repeated measures analysis of variance (time-treatment interaction) with the *Bonferroni* test for *post-hoc* comparisons. Differences in OFT, EPMT, and FST data were analyzed using an independent-samples *t*-test. Prior to analyses, data were checked for conformance to the normal distribution using the Shapiro-Wilk normality test; when normal distribution was not supported, the Wilcoxon rank-sum test was used. A *p* < 0.05 was considered statistically significant. Sham and CCI group ALFF and DC values obtained from fMRI data were compared using two-sample *t*-tests. Results were corrected for multiple comparisons with a combined threshold of a single voxel (*p* < 0.001). AlphaSim estimation was performed using REST v2.329 (http://rfmri.org/dpabi). To decrease the possibility of false-negative results, a threshold (*p* < 0.001, cluster size >12 voxels) was applied to each cohort. Pearson correlation analysis was utilized to evaluate the correlation among behavioral test and fMRI ALFF and DC value data.

## Results

### CCI Induced Mechanical Allodynia

**Figure 2:**
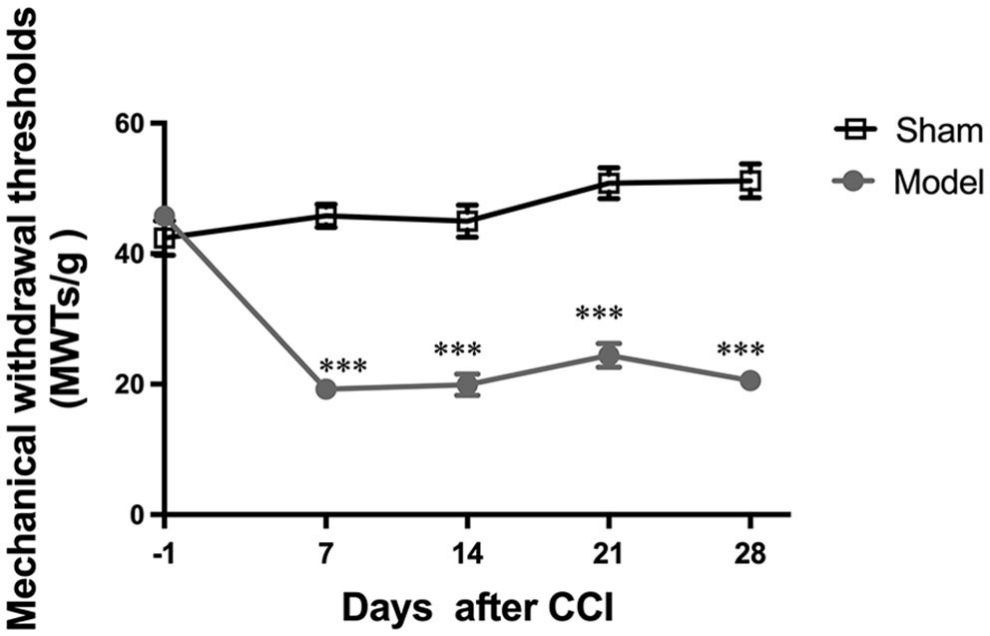
Evaluation of mechanical withdrawal thresholds (MWT). All data are expressed as the Mean ± SEM (*n* = 8 per group). ***p < 0.001 vs. the sham group.

A main effect of the CCI model [*F*_(1, 14)_ = 109.9; *p* < 0.0001] and a significant group × time interaction [*F*_(4, 56)_ = 38.39; *p* < 0.0001] were noted among the two groups. The day before CCI surgery, no significant differences in MWT among the two groups were found (*p* > 0.05). After the surgery, CCI group ipsilateral hind paw MWT values were noted to significantly decrease from days 7 to 28 as compared with sham group values (all *p* < 0.001; Figure 2), indicating that CCI induced persistent mechanical allodynia.

### CCI Induced Emotional Disorders

**Figure 3:**
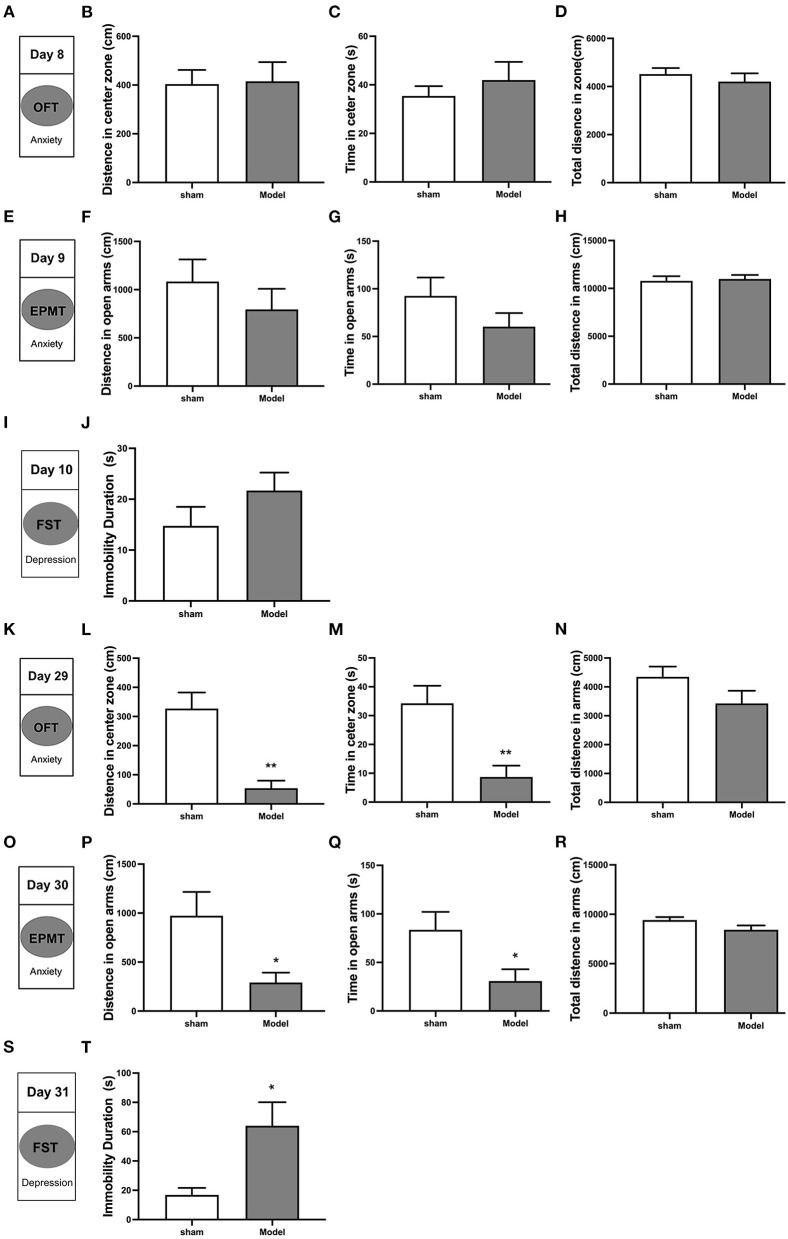
Depressive and anxiety-like behaviors induced by CCI in rats. Anxiety-like behavior was accessed using both the open field test (OFT) **(A–D, K–N)** and the elevated plus maze test (EPMT) **(E–H, O–R)**. Depressive-like behavior was accessed using the forced swimming test (FST) **(I,J,S,T)**. **(A,K)** OFT was performed on days 8 and 29 after CCI surgery; **(B,L)** Distance traveled in the central zone on OFT; **(C,M)** Time spent in the central zone on OFT; **(D,N)** Total distance traveled on OFT; **(E,O)** EPMT was performed on days 9 and 30 after CCI surgery; **(F,P)** Distance traveled in the open arms on EPMT; **(G,Q)** Time spent in the open arms on EPMT; **(H,R)** Total distance traveled on EPMT; **(I,S)** FST was performed on days 10 and 31 after CCI surgery; **(J,T)** Immobility time on FST. All data are expressed as the Mean ± SEM (*n* = 8 per group). *p < 0.05, **p < 0.01 *vs*. sham group.

Behavioral tests conducted on days 8–10 after CCI revealed the following (Figures 3A,E,I): (1) analyses of OFT data revealed no differences in distance traveled (*p* > 0.05) and time spent (*p* > 0.05) in the central zone, as well as total distance traveled (*p* > 0.05) among CCI and sham groups (Figures 3B–D); (2) analyses of EPMT data revealed no differences in distance traveled (*p* > 0.05) and time spent (*p* > 0.05) in maze open arms, and total distance traveled (*p* > 0.05) among the two groups (Figures 3F–H); (3) analyses of FST data revealed CCI group immobility time to be similar to that of the sham group (*p* > 0.05; Figure 3J). Behavioral tests conducted from days 29 to 31 revealed the following: (Figures 3K,O,S) (1) analyses of OFT data revealed that CCI group rats traveled shorter distances (*p* < 0.001) and spent less time (*p* < 0.01) in the central zone as compared to sham group rats (Figures 3L,M). No differences in total distance traveled were noted between groups (*p* > 0.05; Figure 3N); (2) analyses of EPMT data revealed that CCI rats traveled significantly shorter distances (*p* < 0.05) and spent significantly less time (*p* < 0.05) in the open arms of the maze (Figures 3P,Q). The total distance in EPM did not show any difference (*p* > 0.05) in the two groups (Figure 3R); (3) analyses of FST data revealed that CCI rats were immobile for significantly longer as compared to sham rats (*p* < 0.05; Figure 3T). These findings suggest that depressive and anxiety-like behaviors induced by CCI do not appear in the early stages of pathology until approximately 4 weeks after injury.

### Post-CCI Alterations in ALLF at Different Time Points

**Figure 4:**
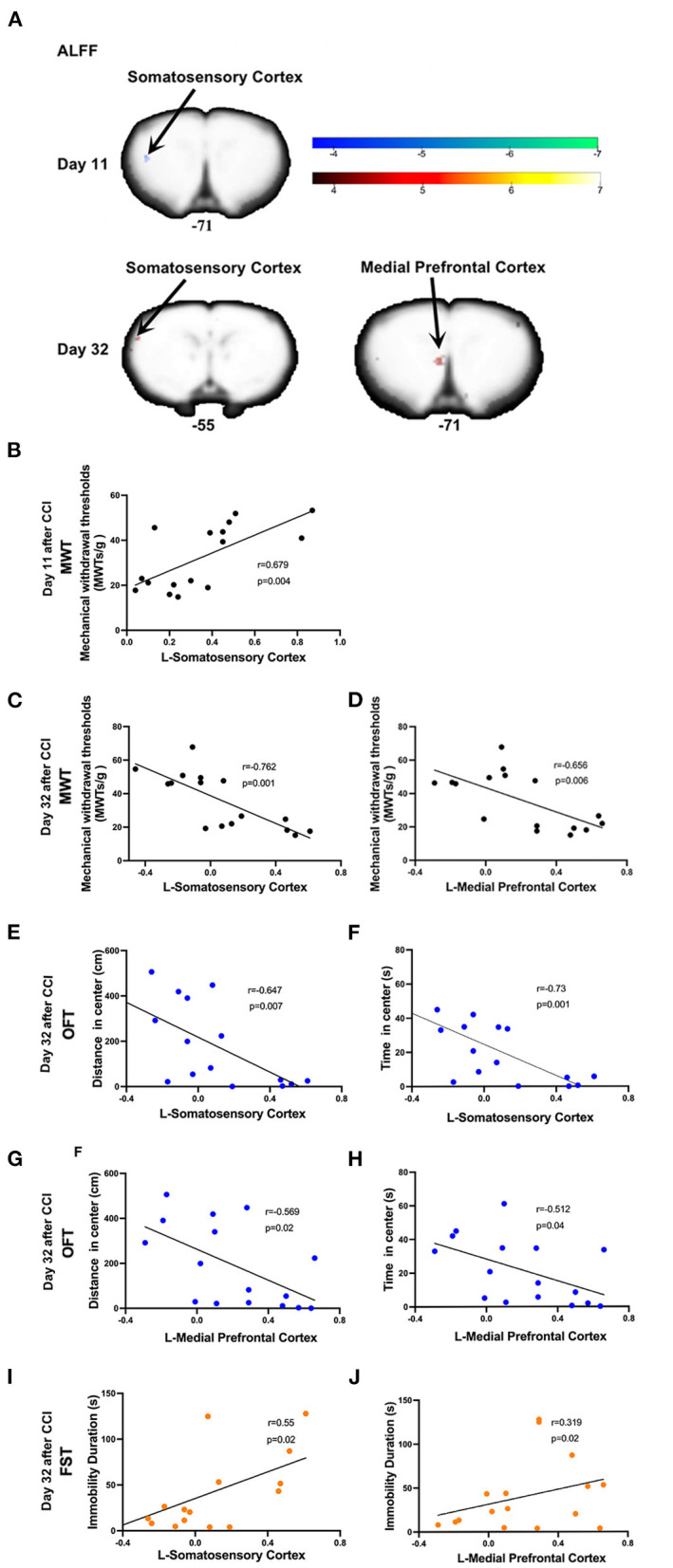
Significant alternations in ALFF induced by CCI and correlations between ALFF values and the mechanical withdrawal thresholds (MWT) values, open field test (OFT) data, and forced swimming test (FST) data. **(A)** CCI group rats were found to exhibit significantly lower ALFF values in the left somatosensory cortex on day 11 after CCI surgery and significantly higher ALFF values in both the left somatosensory cortex and mPFC on day 32 after CCI surgery as compared to sham group rats; **(B)** ALFF values in the left somatosensory cortex (10 days after CCI surgery) positively correlated with MWT values (7 days after CCI surgery); **(C–H)** ALFF values in the left somatosensory cortex and mPFC (32 days after CCI surgery) negatively correlated with MWT (28 days after CCI surgery), distance traveled and time spent in the central zone of OFT (29 days after CCI surgery); **(I,J)** The ALFF values in the left somatosensory cortex and mPFC (32 days after CCI surgery) was positively correlated with immobility time on FST (31 days after CCI surgery).

On day 11 post-CCI, experimental group rats exhibited significantly lower ALFF values in the left somatosensory cortex as compared to sham group rats (Figure 4A and Table 1). At this time point, Pearson correlation analysis revealed a positive correlation between ALFF values in the left somatosensory cortex and MWT values (*r* = 0.679, *p* = 0.004; Figure 4B). On day 32 post-CCI, experimental group rats were found to exhibit significantly higher ALFF values in the left somatosensory cortex and left mPFC as compared to sham group rats (Figure 4A and Table 1). Pearson correlation analysis revealed that both ALFF values in the left somatosensory cortex and left mPFC negatively correlated with MWT values (*r* = −0.762, *p* = 0.001 for the left somatosensory cortex, Figure 4C; *r* = −0.656, *p* = 0.006 for the left mPFC, Figure 4D), distance traveled in the central zone of the maze on OFT (*r* = −0.647, *p* = 0.007 for the left somatosensory cortex, Figure 4E; *r* = −0.569, *p* = 0.02 for the left mPFC, Figure 4F), and length of time spent in the central zone on OFT (*r* = −0.73, *p* = 0.001 for the left somatosensory cortex, Figure 4G; *r* = −0.512, *p* = 0.04 for the left mPFC, Figure 4H). ALFF values in the left somatosensory cortex and left mPFC, however, were both noted to positively correlate with immobility duration values on FST (*r* = 0.55, *p* = 0.02 for the left somatosensory cortex, Figure 4I; *r* = 0.319, *p* = 0.02 for the left mPFC, Figure 4J) throughout the aforementioned time period. No significant correlation between EPMT and ALFF values in the left somatosensory cortex (*r* = −0.4, *p* = 0.08 for distance traveled in the open arms; *r* = −0.41, *p* = 0.1 for time spent in the open arms) and left mPFC (*r* = −0.373, *p* = 0.1 for distance traveled in the open arms; *r* = −0.406, *p* = 0.1 for time spent in the open arms), however, was noted (Supplementary Figure 1).

**Table 1:** Results of amplitude of low-frequency fluctuations (ALFF) analysis.

**Contrast name**				**MNI-coordinates**		
**Time**	**Region label**	**Extent**	***t*-value**	**x**	**y**	**z**
Day 11	L-Somatosensory Cortex	12	−5.324	−46	1	−71
Day 32	L-Somatosensory Cortex	24	6.063	−54	24	−37
	L-Medial Prefrontal Cortex	20	5.741	−7	−5	−69

*L, left; R, right*.

### Post-CCI Alterations in DC at Different Stages

**Figure 5:**
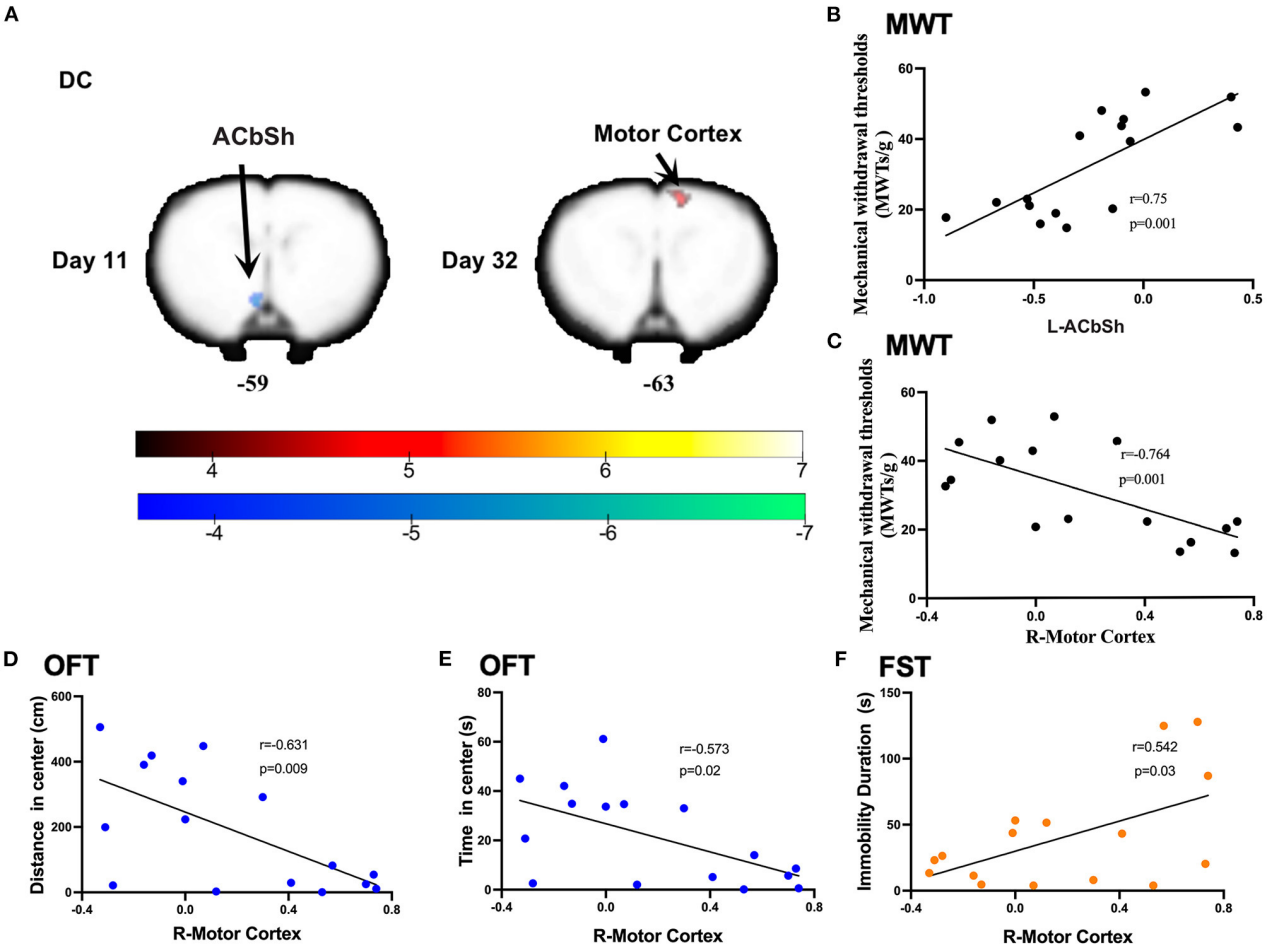
Significant DC alternations induced by CCI and correlations between DC values and the mechanical withdrawal thresholds (MWT), open field test (OFT), and forced swimming test (FST) data. **(A)** CCI group rats exhibited significantly lower DC values in the left nucleus accumbens shell (ACbSh) on day 11 after CCI surgery and significantly higher DC values in the right motor cortex on day 32 after CCI surgery as compared to sham rats; **(B)** DC values in the ACbSh (10 days after CCI surgery) positively correlated with MWT values (7 days after CCI surgery); **(C–E)** DC values in the right motor cortex (32 days after CCI surgery) negatively correlated with MWT values (28 days after CCI surgery), distance traveled and time spent in the central zone of OFT (29 days after CCI surgery); **(F)** DC values in the right motor cortex (32 days after CCI surgery) positively correlated with the immobility time on FST (31 days after CCI surgery).

Compared to sham rats, CCI rats were found to exhibit significantly decreased DC values in the left nucleus accumbens shell (ACbSh) on day 11 post-CCI and the right motor cortex on day 32 post-CCI (Figure 4A and Table 2). Pearson correlation analysis revealed that left ACbSh DC values positively correlated with MWT values on day 11 post-CCI (*r* = 0.75, *p* = 0.001, Figure 5B) and that right motor cortex DC values negatively correlated with MWT values on day 11 post-CCI (*r* = −0.764, *p* = 0.001, Figure 5C). Right motor cortex DC values were similarly found to negatively correlate with values of distance traveled within the central zone of the maze (*r* = −0.631, *p* = 0.009, Figure 5D) and time spent in the central zone (*r* = −0.573, *p* = 0.02, Figure 5E) on OFT, and positively correlate with immobility duration values on FST (*r* = −0.542, *p* = 0.03, Figure 5F). No significant correlation between right motor cortex DC values and EPMT data were noted (Supplementary Figure 2).

**Table 2:** Differences in DC values between model and sham groups.

**Contrast name**				**MNI-coordinates**		
**Time**	**Region label**	**Extent**	***t*-value**	**x**	**y**	**z**
Day 11	L- nucleus accumbens shell	50	−5.281	−7	−28	−59
Day 32	R- motor cortex	45	5.030	−14	34	−63

*L, left; R, right*.

## Discussion

This study aimed to investigate alterations in brain functional features throughout the development and progression of NeuP. We confirmed that the robust mechanical allodynia occurred early in injury and persisted in a CCI rat model of NeuP. Pain-related negative emotions did not manifest until 4 weeks after nerve injury. ALFF and DC values were found to be significantly decreased in the left somatosensory cortex and ACbSh early in CCI rats as compared to sham controls. When anxiety- and depression-like behaviors manifested, ALFF and DC values were noted to significantly increase in the left somatosensory cortex, left mPFC, and right motor cortex among CCI rats.

As ALFF reflects the magnitude of spontaneous neuronal activity, an altered ALFF suggests abnormal regional neuronal activity. We found that neuronal activity within the somatosensory cortex of CCI group rats is suppressed early after injury yet enhanced in later stages as compared with the control group rats. The somatosensory cortex (primary (S1) and secondary (S2) somatosensory cortices) is responsible for the sensory-discriminative dimension of pain processing and receives noxious somatosensory input from the somatosensory thalamus, coding spatial, temporal, and intensive aspects of noxious somatosensory stimuli (35–38). Several clinical and experimental lines of evidence confirmed that NeuP induces changes in both morphology and plasticity within the somatosensory cortex (39–41). Patients suffering from NeuP display changes in somatosensory cortex activity and anatomy not seen in patients suffering from non-neuropathic chronic pain (42). A longitudinal study reported that lower somatosensory cortex excitability among patients in the acute stages of low back pain was associated with significantly increased odds of developing chronic pain by 6-month follow-up (43). Furthermore, activation of inhibitory neural circuits within the somatosensory cortex using electroacupuncture was reported to alleviate hyperalgesia in the setting of NeuP (44). Interestingly, somatosensory cortex functions are not restricted to pain sensation; rather, they are involved in the regulation of comorbid anxiety in the setting of persistent pain as well as aversive responses to pain (45, 46). Our results supported these concepts and suggested that changes in ipsilateral somatosensory cortex excitability play active roles in the chronification of NeuP and manifestation of negative emotions among CCI model rats with left-sided sciatic nerve ligation. Unlike the left somatosensory cortex, ALFF remained static in the left mPFC and exhibited significant increases only in the later stages of NeuP, also correlating with mechanical hyperalgesia and the manifestation of negative emotions. The mPFC has previously been extensively implicated in the affective emotional and cognitive aspects of pain and pain modulation (47, 48). A study evaluating human fMRI data that analyzed 270 participants across 18 studies found that pain representations were localized to the anterior midcingulate cortex, negative emotion representations to the ventromedial prefrontal cortex, and cognitive control representations to portions of the dorsal midcingulate cortex (49). Many studies have similarly shown that mPFC activity to be altered in the setting of chronic pain (50). Importantly, gray matter mPFC volume decreases among people suffering from chronic pain (51). Layer- and subregion-specific electrophysiological and morphological changes in the mPFC have further been reported in the setting of NeuP (52). The plasticity of serotonin transmission in the mPFC facilitates the manifestation of anxiety associated with NeuP (53). Likewise, the excitability of pyramidal mPFC neurons was noted to increase when depression manifested. Among NeuP rats; this was induced *via* nerve-sparing injury (54). As such, the mPFC plays a key role in the development of negative emotions associated with NeuP. Pain-related neural activity shifts in focus from the acute pain circuit to the emotional one, suggesting an alteration in perception to be a key consequence of NeuP (55).

The evaluation of DC reveals the importance of a neural node within connectivity networks at the voxel level (34). In this study, the left ACbSh was found to exhibit decreased DC early in NeuP. One recent fMRI study reported that patients suffering chronic pain exhibited smaller nucleus accumbens volumes and loss of power in the slow-5 frequency band (0.01–0.027 Hz) only after the onset of the chronic pain phase, highlighting a likely signature of the state of chronic pain (56). As a subregion of the nucleus accumbens, the ACbSh also plays an important role in mediating NeuP (57). Spinal nerve ligation was recently reported to decrease the relative intensity of excitatory and inhibitory synaptic inputs to medium spiny neurons within the ACbSh, resulting in a decreased the frequency of spontaneous inhibitory postsynaptic currents as well as both the frequency and amplitude of spontaneous excitatory postsynaptic currents in medium spiny neurons (58). Here, we found increased DC values in the right motor cortex later in NeuP when anxiety- and depression-like behaviors were noted. In agreement with our findings, another study reported excitability of the motor cortex to be higher in chronic pain patients and possess a significantly negative correlation with anxiety (59). A double-blind randomized study found that 40% of NeuP patients are able to achieve remission with the use of motor cortex stimulation *via* surgically implanted electrodes (60). A systematic review and meta-analysis of fMRI studies also found that the motor cortex of such patients exhibited significantly decreased activity after treatments as compared to baseline (40).

The somatosensory cortex, mPFC, nucleus accumbens, and motor cortex likely possess functional connections that participate in pain modulation. For instance, the density of perineuronal nets in the somatosensory cortex and mPFC were found to be enhanced in a mouse model of chronic inflammatory pain (61). Motor cortex stimulation was found to block the transmission of somatosensory information to the primary somatosensory cortex and alleviate chronic pain (62). Corticotropin-releasing factor neurons of the mPFC project directly to the nucleus accumbens and increased activity in these neurons in the setting of NeuP facilitates behavioral responses to morphine reward (63). Interestingly, the practice of traditional mindful breathing was found to successfully modulate functional connectivity between the prefrontal and primary somatosensory cortices and relieve pain (64).

This study was not without limitations. Here, we only focused on evaluating functional changes in brain regions as NeuP developed but did not investigate for structural abnormalities. Further studies are needed to elucidate the contribution of these brain structures to pain sensitivity and the manifestation of negative emotions. In addition, future study of changes in brain plasticity is warranted. Finally, patient sex differences were not considered in this study. A clear sexual dimorphism in NeuP is known to exist, with females reported to be more sensitive (65).

In conclusion, our study indicates that longitudinal changes in intrinsic brain activity are associated with the development of NeuP. More specifically, we found lower neuronal activity and voxel-voxel connectivity in the somatosensory cortex and ACbSh to be key in nociception and the modulation of pain processing early in NeuP when maximal allodynia was apparent. With the manifestation of depressive and anxiety-like behaviors, higher neuronal activity and voxel-voxel connectivity in the somatosensory cortex, mPFC, and motor cortex highlight the key role of these regions in the modulation of negative emotions. This study provides a basis for future investigation aiming to advance neuromodulatory intervention for NeuP.

## Data Availability Statement

The original contributions presented in the study are included in the article/Supplementary Material, further inquiries can be directed to the corresponding authors.

## Ethics Statement

The animal study was reviewed and approved by Institutional Animal Care Committee of Yueyang Hospital of Integrated Traditional Chinese and Western Medicine, Shanghai, China's Shanghai University of Traditional Chinese Medicine.

## Author Contributions

KW and X-YH conceived and designed the experiments. Y-NZ and X-XX performed the experiments and analyzed the data. LC, XD, and H-TP helped with the behavior test experiments. KW, X-YH, and Y-NZ wrote and modified the manuscript. All authors discussed the results and reviewed the manuscript.

## Funding

The project was funded by the National Natural Science Foundation of China (81973940, 81673756), Accelerating the Development of Chinese Medicine Three-Year Action Plan of Shanghai [no. ZY (2018-2020)-CCCX-2004-04], the Clinical Key Specialty Construction Foundation of Shanghai (no. shslczdzk04701), and the Shanghai Clinical Research Center for Acupuncture and Moxibustion (20MC1920500).

## Conflict of Interest

The authors declare that the research was conducted in the absence of any commercial or financial relationships that could be construed as a potential conflict of interest.

## Publisher's Note

All claims expressed in this article are solely those of the authors and do not necessarily represent those of their affiliated organizations, or those of the publisher, the editors and the reviewers. Any product that may be evaluated in this article, or claim that may be made by its manufacturer, is not guaranteed or endorsed by the publisher.

## Supplementary Material

Supplementary Figure 1 **(A)** The ALFF values in the left mPFC (32 days post-CCI surgery) negatively correlated with MWT values (28 days post-CCI surgery); **(B–E)** No significant correlations were observed between ALFF values in the left somatosensory cortex and mPFC (32 days after CCI surgery) and distance traveled and time spent in open arms on EPMT (29 days post-CCI surgery) (all *p* > 0.05).

Supplementary Figure 2 No significant correlations were observed between DC values in the right motor cortex (32 days post-CCI surgery) and distance traveled **(A)** and time spent **(B)** in the open arms on EPMT (29 days post-CCI surgery) (all *p* > 0.05).
