# Supplementary figures and images for: Brain Functional Alteration at Different Stages of Neuropathic Pain With Allodynia and Emotional Disorders
Source: Front Neurol. 2022 May 2;13:843815. doi: 10.3389/fneur.2022.843815 (PMC9108233; doi:10.3389/fneur.2022.843815)

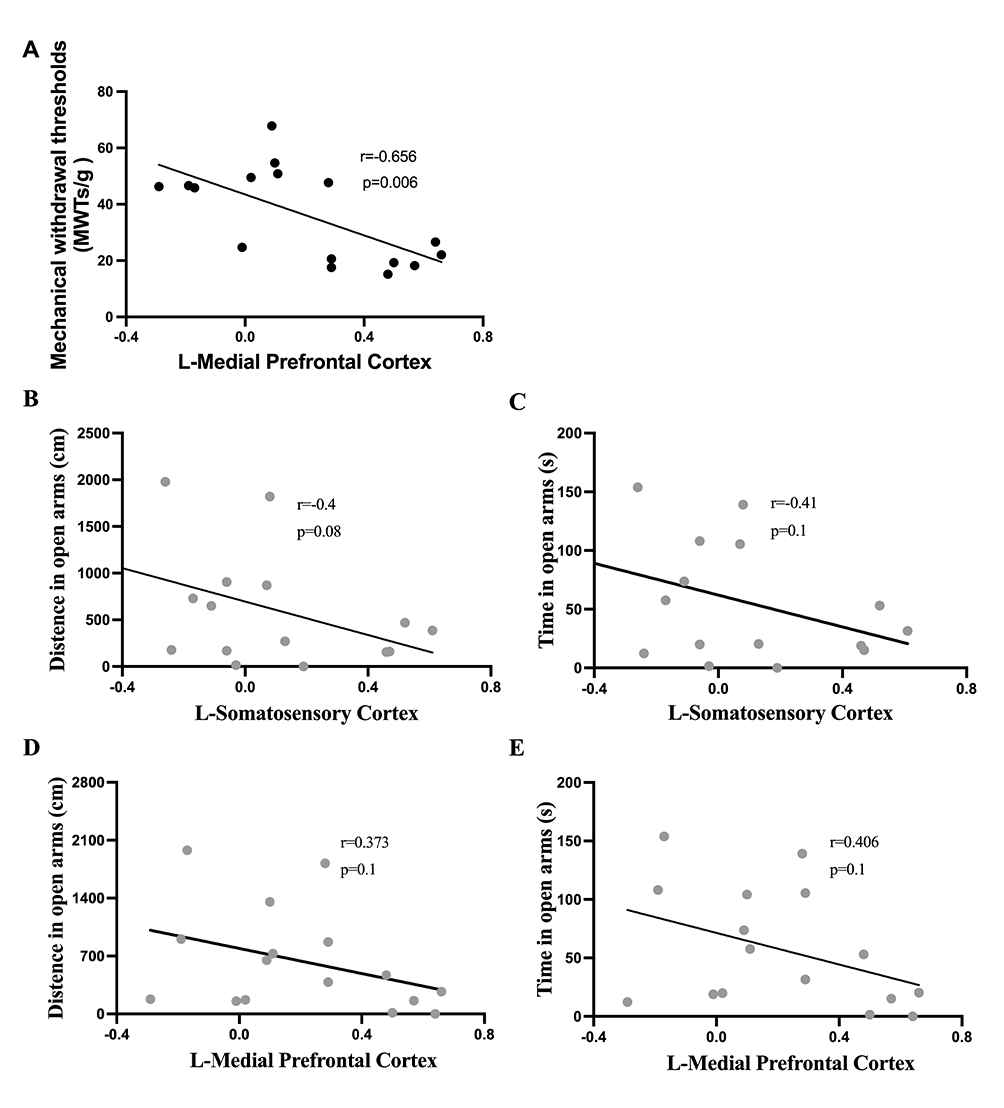

Supplement: Supplementary Figure 1 — (A) The ALFF values in the left mPFC (32 days post-CCI surgery) negatively correlated with MWT values (28 days post-CCI surgery); (B–E) No significant correlations were observed between ALFF values in the left somatosensory cortex and mPFC (32 days after CCI surgery) and distance traveled and time spent in open arms on EPMT (29 days post-CCI surgery) (all p > 0.05). [file Image_1.TIF]

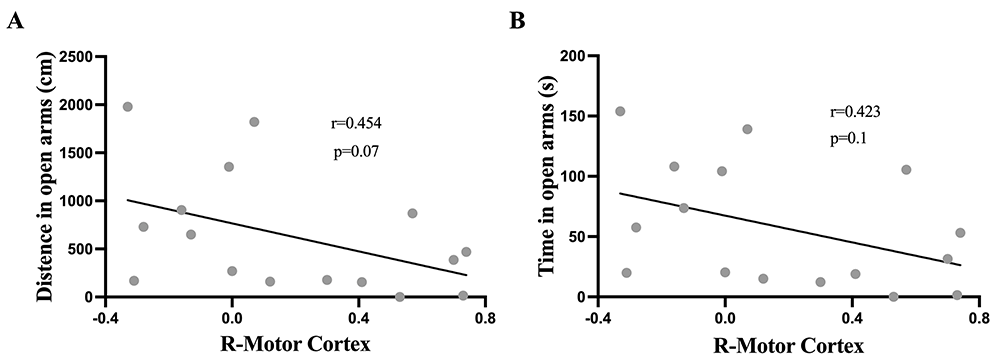

Supplement: Supplementary Figure 2 — No significant correlations were observed between DC values in the right motor cortex (32 days post-CCI surgery) and distance traveled (A) and time spent (B) in the open arms on EPMT (29 days post-CCI surgery) (all p > 0.05). [file Image_2.TIF]
